# Continuously active disinfectant inactivates severe acute respiratory coronavirus virus 2 (SARS-CoV-2) and human coronavirus 229E two days after the disinfectant was applied and following wear exposures

**DOI:** 10.1017/ice.2021.481

**Published:** 2021-12-02

**Authors:** William A. Rutala, Luisa A. Ikner, Curtis J. Donskey, David J. Weber, Charles P. Gerba

**Affiliations:** 1 Division of Infectious Diseases, University of North Carolina School of Medicine, Chapel Hill, North Carolina; 2 Department of Environmental Science, University of Arizona, Tucson, Arizona; 3 Infectious Disease Section, Cleveland VA Medical Center, Cleveland, Ohio; 4 Hospital Epidemiology, University of North Carolina Medical Center, Chapel Hill, North Carolina

## Abstract

The surface environment in rooms of coronavirus disease 2019 (COVID-19) patients may be persistently contaminated despite disinfection. A continuously active disinfectant demonstrated excellent sustained antiviral activity following a 48-hour period of wear and abrasion exposures with reinoculations. Reductions of >4-log_10_ were achieved within a 1-minute contact time for severe acute respiratory coronavirus virus 2 (SARS-CoV-2) and the human coronavirus, 229E.

Hospital-room environmental surfaces and noncritical medical devices are frequently contaminated, and they serve as a source of healthcare pathogens including severe acute respiratory syndrome coronavirus 2 (SARS-CoV-2). Although indirect transmission via fomites is not thought to be the primary way the virus spreads, the role of the contaminated healthcare environment in the transmission of SARS-CoV-2 among patients and/or healthcare personnel remains unclear. In laboratory testing, SARS-CoV-2 has been shown to remain viable on surfaces for hours to days.^1^ The surface environment in rooms of coronavirus disease 2019 (COVID-19) patients may be persistently contaminated with SARS CoV-2 RNA despite routine room cleaning and disinfection.^2^

We recently reported on a novel continuously active disinfectant against pathogens causing healthcare-associated infections [eg, methicillin-resistant *Staphylococcus aureus* (MRSA), vancomycin-resistant *Enterococcus* spp (VRE), *Candida auris*] and multidrug-resistant organisms (MDROs) [eg, carbapenem-resistant *Klebsiella pneumoniae*].^3,4^ The application of this continuously active disinfectant on portable equipment resulted in significant reductions in aerobic colony counts over 7 days, and in lower recoveries of *S. aureus* and enterococci.^4^ In the present study, we evaluated the residual efficacy of a continuously active disinfectant that is registered by the Environmental Protection Agency (EPA) to kill SARS-CoV-2 and human coronavirus 229E (HCoV-229E) on surfaces for 48 hours.

## Methods

We investigated the continuously active disinfectant against HCoV-229E and SARS-CoV-2 using the EPA Protocol #01-1A “Protocol for Residual Self-Sanitizing Activity of Dried Chemical Residuals on Hard, Non-Porous Surfaces,” with modifications for viruses.^5^ The method simulates dry and wet wiping by incorporating “wear” of the test surface as well as reinoculations of the test and control surfaces over a period of at least 24 hours following product application. Glass surfaces (2.5 cm × 2.5 cm) were initially inoculated with ≥5-log_10_ of virus per carrier, treated with the novel disinfectant (3 sprays, 15.25–20.3 cm from the surface), and allowed to dry overnight. The carriers were abraded using a standardized abrasion machine (Gardco Model D10V, Paul N. Gardner Co, Pompano Beach, FL) under multiple alternating dry and wet wiping conditions (6 dry cycles, 6 wet cycles, total 12 cycles [2 passes per cycle = 24 passes] interspersed with 6 reinoculations with ≥3-log_10_ of the test pathogen. The mean log_10_ viral titer of the initial viral inoculum volume per carrier (0.010 mL) for SARS-CoV-2 was 5.67 ± 0.17, and the reinoculation titers were 3.67 ± 0.17 on day 1 and 3.92 ± 0.09 on day 2 (n = 2). The mean log_10_ viral titer of the initial viral inoculum volume per carrier (0.010 mL) for HCoV-229E was 5.63 ± 0.18, and the reinoculation titers were 4.00 ± 0.35 and 3.75 ± 0.00 on day 1 and day 2 (n = 2), respectively. After the 48-hour period of wear exposures, the surfaces were reinoculated with ≥5-log_10_ of virus to assess the sanitizing efficacy of the continuously active disinfectant to kill >99.9% following a 1-minute contact time. All viral preparations were prepared with fetal bovine serum to achieve a 5% organic soil load. Carriers were neutralized using 1 mL Letheen broth base (Neogen, Lansing, MI) followed by immediate passage through a Sephadex G-10 gel filter column via centrifugation (3,500 ×*g* for 5 minutes).

Human coronavirus 229E (ATCC VR-740), an enveloped respiratory virus, was procured from the American Type Culture Collection (ATCC, Manassas, VA). Propagation and assay of HCoV-229E was performed using the human lung fibroblast MRC-5 cell line (ATCC CCL-171). The SARS-CoV-2 isolate USA-WA1/2020 was deposited by the Centers for Disease Control and Prevention and was obtained through BEI Resources, the National Institute of Allergy and Infectious Diseases, and the National Institutes of Health. SARS-CoV-2 (BEI NR-52281) was propagated and assayed using the Vero E6 cell line (ATCC CRL-1586). HCoV-229E and SARS-CoV-2 viral stocks were enumerated on their respective host cell lines seeded into 96-well cell culture trays using the TCID_50_ technique.

The continuously active disinfectant is EPA-registered as Firebird F130 (Microban Products, Huntersville, NC) and is marketed as Sani-24 by Professional Disposable International (Woodcliff Lake, NJ). The product has a disinfectant claim against 32 microorganisms and a residual claim against 5 bacteria.

## Results and Discussion

The continuously active disinfectant studied demonstrated excellent sustained antiviral activity (>4.0-log_10_ reduction) within 1 minute against human coronavirus 229E and SARS-CoV-2 prepared with 5% organic soil following a 48-hour period of wear and abrasion exposures (Table 1). We detected no reduction in viral titer for the control.

**Table 1.** Inactivation of SARS-CoV-2 and the Human Coronavirus 229E by a Continuously Active Disinfectant Following a 48-Hour Period of Wear and Abrasion Exposures

Carrier Treatment with Wears and Reinoculations	HCoV 229E Mean Viral Recovery per Carrier (Log_10_ ± S.D.)	SARS-CoV-2 Mean Viral Recovery per Carrier (Log_10_ ± S.D.)	HCoV 229E Log_10_ Reduction	SARS-CoV-2 Log_10_ Reduction
Control (sterile NP water, n=3)	6.00 ± 0.25	5.72 ± 0.08	NA	NA
Continuously acting disinfectant (n=3)	≤1.50 ± 0.00	≤1.50 ± 0.00	≥4.50	≥4.22

Notes: Contact time = 1 minute; NA = not applicable.

Environmental contamination plays an important role in the transmission of several key healthcare-associated pathogens, including MRSA, VRE, and MDROs. Evidence in the literature supporting the role of the contaminated surface environment in the transmission of healthcare pathogens has been published.^6^ Many of the studies demonstrated that rooms are not adequately cleaned or disinfected, and patients admitted to a room previously occupied by a patient colonized or infected with a pathogen (eg, MRSA, VRE, *Clostridioides difficile*) have an increased likelihood of developing colonization or infection with that pathogen.^7^ To minimize this risk, improved terminal room decontamination (eg, supplemental ultraviolet disinfection following cleaning and disinfection) of contact precaution patient rooms has led to a decreased rate of infection in patients subsequently admitted to the room where the prior occupant was colonized or infected.^8^ However, the limitation of these “no touch” technologies is that currently they can only be used for terminal room disinfection (ie, not daily cleaning and disinfection) because they require removal of the patients, visitors, and healthcare personnel from the room.

Because routine cleaning and disinfection of room surfaces by environmental services is frequently inadequate^7^ and surfaces rapidly become recontaminated^9^ by patients, visitors, and staff, continuous room decontamination methods are being evaluated.^10^ These findings highlight the potential to interrupt transmission from contaminated surfaces via healthcare provider’s hands by suboptimal compliance with hand hygiene or inappropriate glove use.

A continuously active disinfectant is a continuous room decontamination method.^10^ That is, if an antimicrobial residue was left on a disinfected surface and it persists on the surface for ≥24 hours, it could reduce or eliminate the problem of continuous recontamination and minimize the role of environmental surfaces as reservoirs of pathogens by eliminating them on the treated surface. The intent of this technology is to make surfaces hygienically clean (not sterile), or free of pathogens in sufficient numbers to prevent human disease. In this study, we subjected a continuously active disinfectant to wear and abrasion exposures over 48 hours to assess residual antiviral efficacy against SARS-CoV-2 and HCoV-229E on surfaces for ≥24 hours.

Previous studies demonstrated persistent antimicrobial activity (ie, 3–5-log_10_ reduction) 24 hours after application for many healthcare pathogens within a contact time of 5 minutes.^3,4^ In this study, we have demonstrated residual efficacy of the continuously active disinfectant to inactivate SARS-CoV-2 and HCoV-229E within 1 minute following 12 cycles of alternating dry and wet abrasions (6 dry and 6 wet) performed with reinoculations during the 48 hours after the product application. Based on our data using SARS-CoV-2 as well as studies with several common healthcare pathogens (eg, MRSA, VRE), continuously active disinfectants can significantly reduce bacterial, viral, and yeast populations that contact treated surfaces within 1–5 minutes over ≥24 hours.^3,4^ If the microbial load on surfaces is pathogen free or pathogens are significantly reduced, the treated environmental surface will not act as a reservoir or source for pathogens (including SARS-CoV-2) linked to disease transmission.
